# Antibiotic prophylaxis in primary and revision shoulder replacement: a systematic review

**DOI:** 10.1186/s12891-020-03332-z

**Published:** 2020-05-11

**Authors:** Umile Giuseppe Longo, Vincenzo Candela, Gabriella Facchinetti, Anna Marchetti, Silvia Dsoke, Claudia Mazzella, Laura Risi Ambrogioni, Maria Grazia De Marinis, Vincenzo Denaro

**Affiliations:** 1 Department of Orthopaedic and Trauma Surgery, Campus Bio-Medico University, Via Alvaro del Portillo, 200, 00128 Trigoria, Rome, Italy; 2 Research Unit Nursing Science, Campus Bio-Medico di Roma University, Rome, Italy

**Keywords:** Shoulder arthroplasty, Total shoulder replacement, Antibiotic prophylaxis, Infection, *Propionibacterium acnes*

## Abstract

**Background:**

One of the most common bacteria responsible for most Periprosthetic joint infection (PJI) is *Propionibacterium acnes*. Even though the rate of infections in patients undergoing total shoulder arthroplasty is increasing, effective diagnostic tests and the precautions taken during the surgery are not yet adequate. This systematic review aims to evaluate the effectiveness of antimicrobial prophylaxis in PJI in shoulder replacement and to provide health workers with the best approach to the use of antimicrobial agents based on currently available clinical evidence.

**Methods:**

a systematic review of the literature was carried out in accordance with the PRISMA Statement. Studies concerning the effectiveness of antimicrobial prophylaxis in the prevention of PJI in patients undergoing shoulder replacement were included.

**Results:**

Seven studies were included in the final analysis because they were considered valid. A total of 3272 patients underwent a surgical procedure, most of which were males. The male population has a greater presence of hair, therefore a greater risk of P. acnes. in surface cultures. Patients were assessed at an average follow-up period of 20 months ranging from 9 weeks to 53 months.

**Conclusion:**

The optimal perioperative antimicrobial regimen is controversial. The clinical guidelines recommend the use of only one antibiotic as prophylaxis but considering the increase in the rates of antibiotic-resistant infections, the question arises whether antibiotic prophylaxis should be extended for adequate coverage. Shoulder arthroplasty performed on the male population must be carefully checked after surgery for the possible presence of P. Acnes.

## Background

Given the high success rate, total shoulder arthroplasty has become an effective surgical procedure for glenohumeral osteoarthritis. However, periprosthetic joint infection (PJI) still remains a devastating complication that negatively affects the overall outcome [1–6]. Furthermore, the PJI is not only a catastrophic clinical failure, but it also represents an important burden for the health care system that requires long therapeutic support and expensive revision interventions [7].

The most common bacteria responsible for most PJIs are *Propionibacterium acnes (P. acnes), Staphylococcus aureus, Staphylococcus epidermidis* and coagulase-negative Staphylococcus [8, 9]. In particular, the *P. acnes* is a gram-positive micro-organism present in the dermis and sebaceous glands of most individuals [6, 10]. In the axilla area, there are numerous sebaceous glands which, by secreting the sebum that protects and lubricates the skin, also offer the opportunity for *P. acnes* to grow and contaminate the shoulder area [11].

PJIs after total shoulder arthroplasty, especially in patients with *P. acnes*, are more challenging than those occurring in other joints [12]. In particular, *P. acnes* is characterized by low virulence and, therefore, the laboratory tests normally used for early diagnosis (C reactive protein and red blood cells, white blood cells, erythrocyte sedimentation rate) appear normal even though the infection is in progress. The atypical characteristics of this bacterial strain explain why the diagnosis of infection is often only possible at the time of revision of the prosthesis when intraoperative cultures are performed.

Even though the rate of infections in patients undergoing total shoulder arthroplasty is increasing, effective tests for diagnosis have not yet been discovered [13]. Therefore, in order to reduce contamination of PJI preventive measures are to be improved, this to guarantee patients health, safety and to optimize forecasts prior to surgical intervention. However, the precautions taken at the time of surgery, such as intravenous antibiotics and standard skin preparation solution, are not yet sufficiently adequate to ensure sterilization of the incision area [11, 14]. Therefore, the surgeon should avoid contact with the skin to minimize the risk of infection [15–17].

This systematic review aims to evaluate the suitability of antimicrobial prophylaxis in prevention of PJI in shoulder replacement and to provide health workers with the best suggestion for the use of antimicrobial agents based on currently available clinical evidence.

## Methods

This systematic review was performed in accordance with the Preferred Reported Items of Systematic Review and Meta-analysis Statement (PRISMA) [18].

### Eligibility criteria

Studies concerning the effectiveness of antimicrobial prophylaxis in the prevention of PJI in patients undergoing shoulder replacement were taken into account. Inclusion criteria were as follows: shoulder replacement surgery; PJI rate; mode and dosage of the antibiotics used. Missing data on these parameters warranted the exclusion from this systematic review. According to the Oxford Center of EBM, all articles of level I-IV, regardless of the country where they were conducted, were eligible for inclusion in the review. To qualify for the study, articles had to be published in a peer-reviewed journal. This systematic review does not include systematic reviews, animal studies, cadaver or in vitro investigations, clinical cases, technical notes, biomechanical reports, educational courses and letters to publishers.

### Search strategy

An overall search of the PubMed, Medline, CINAHL, Embase, Cochrane, Google Scholar, and Ovid databases was performed using the following combinations of the keywords: “arthroplasty, shoulder”, “total shoulder replacement”, “shoulder replacement arthroplasty”, “total shoulder arthroplasty”, “infected shoulder arthroplasty”, “infected shoulder replacement”, “prosthetic infection”, “antibiotic prophylaxis”, “antibiotic premedication”, “antimicrobial prophylaxis” “infection”, “*Propionibacterium acnes*”. We selected articles published from the inception of the database to 18 February 2019. Also, a reference list of guidelines of International Consensus Meeting (ICM) on musculoskeletal infection ICM Philly Part III Shoulder (https://icmphilly.com) was consulted to extend even more the directives concerning the review’ topic. Cross-searches were made to obtain relevant and valid articles for the study.

### Data extraction

The search was conducted separately by three independent reviewers (SD, CM, VC). In the first phase, all the articles were checked for relevance, through titles and abstracts. In the second phase, the selected abstracts were screening by full-text. To minimize selection bias and errors, the three investigators (SD, CM, VC) separately examined the abstract of each publication. Finally, all articles selected and excluded from the study, including their reference lists, were discussed by all authors to reach an agreement. Potential disagreement among investigators regarding the inclusion and exclusion criteria were resolved by the senior investigator (V.D.) that took the final decision. A meta-analysis could not be performed due to the limited data available. A narrative synthesis was carried out to determine and analyze the tests and the best practice for the effectiveness of antibiotics in the prevention of PJI in patients undergoing shoulder replacement.

### Quality assessment

All included studies in the systematic review were assessed using the “Quality Assessment Tool for Quantitative Studies”, a standardized table to appraise study quality with respect to sources of bias. This standardized tool was developed to provide high-quality systematic reviews and evidence to support practice. Six sections were recorded: selection bias; confounders; study design; data collection methods; blinding; withdrawals and dropouts. These dominoes can be rated as ‘strong’, ‘moderate’ or ‘weak’. Studies classified as weak on at least two domains are assigned an overall score of “weak”, while if they do not have “weak” they are considered “strong”.

## Results

**Fig. 1:**
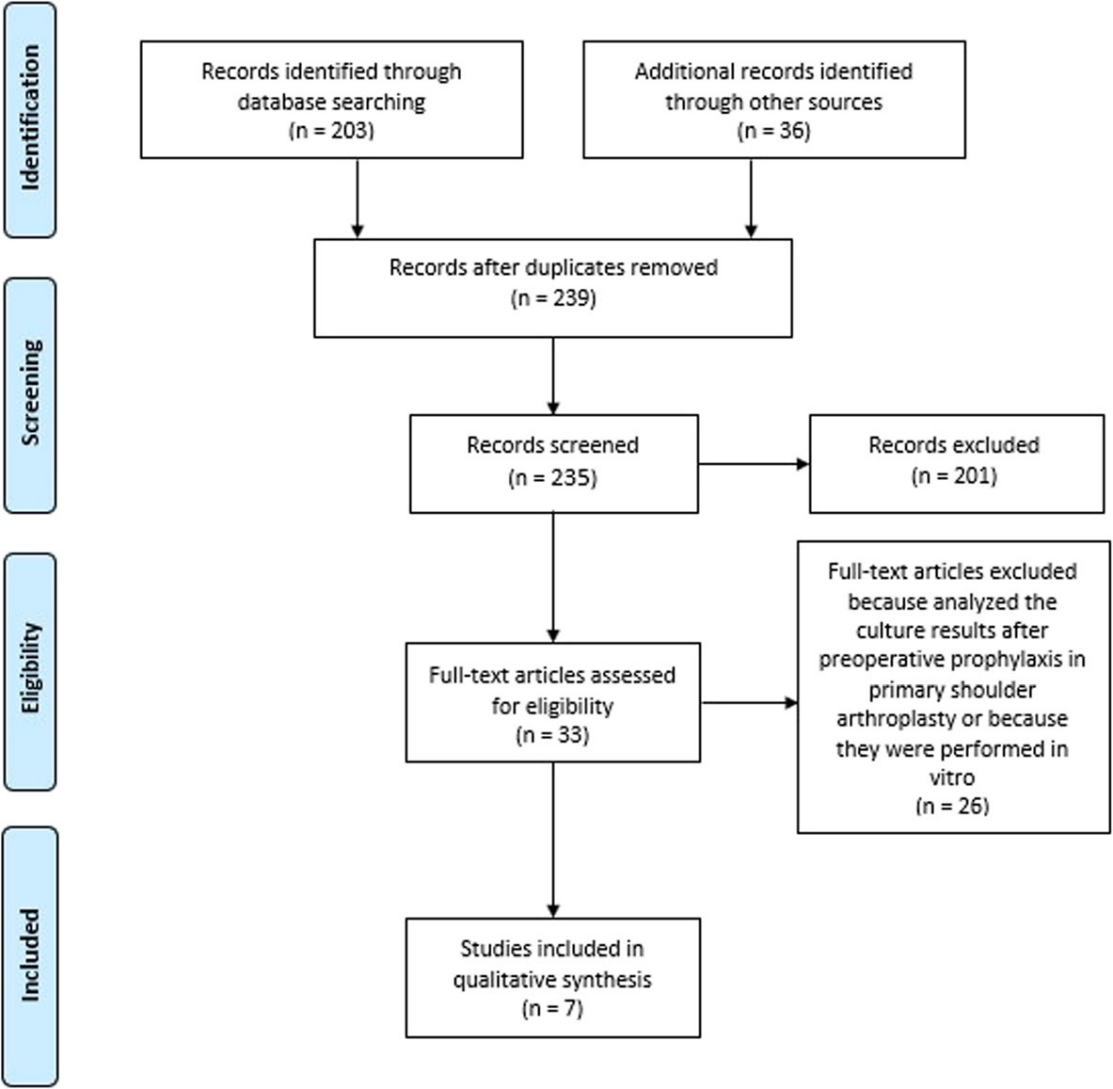
Prisma Flow Diagram

The articles selection process is illustrated in Fig. 1.

Searches yielded a total of 239 articles. Additional records identified 10 guidelines and 33 articles in cross-reference search. After duplicate removal and titles, abstracts review, 9 studies reported considerable information about antimicrobial prophylaxis in shoulder replacement. Two of the 9 articles were excluded after a careful reading because the first analyzes the culture after pre-operative prophylaxis in shoulder arthroplasty, the second analyzes the results in vitro*.* The last 7 studies were included in the final analysis because they were considered suitable for the three reviewers.

### Study characteristics

The search strategy yielded studies from 1995 until 2015.

The study characteristics extracted from the seven selected studies (author, publication date, the study design, patient assignment, the age range, the duration of the study and outcomes measured) are summarized in Table 1. Due to the different inclusion criteria and design of the studies, high clinical heterogeneity was found between the studies. A total of 3272 patients underwent a surgical procedure, most of which were males in 3 of the studies examined [19, 21, 23] with a mean age of 42.8. From superficial cultures of P. *acnes,* it was found that in the male population there were higher rates due to the presence of hair [20] Patients were assessed at an average follow-up period of 20 months, ranging from 9 weeks [20] to 53 months [21].

**Table 1:** Characteristics of included studies

Author	Year	Study design (Oxford Center of EBM level)	Population	Patients with ATB therapy	Shoulder Involved	Age (mean)	Gender (m/f)	Microorganism	Type of infection (a/c) *	Time of follow up	Antibiotic
***Pérez-Prieto[***19***]***	2016	Prospective randomized and controlled study (I)	28	28	3	74	15/13		A = 14; C = 14		Cefalosporin Glycopeptide
***Kong Koh,[***20***]***	2016	Randomized study (I)	30	30	30	74	7/23	P. acnes		9- months	Cefazolin Gentamicin
***Lutz[***21***]***	2005	Retrospective study II	52	35	15	51,8	37/15	P. acnes		average follow-up 37.1 months	Clindamycin–Ofloxacin (*n* = 14PZ) Rifampicin–Ofloxacin(*n* = 6PZ) Clindamycin–Rifampicin (*n* = 3PZ) Cloxacillin–Ofloxacin(*n* = 2) Rrifampicin–Pristinamycin (*n* = 2PZ) Ceftriaxone–pristinamycin(*n* = 1PZ) Cefepime–Ofloxacin (n = 1PZ) eicoplanin–Rifampicin (n = 1PZ) Clindamycin–Fusidic Acid (*n* = 1PZ) Clindamycin monotherapy(n = 1PZ) Vancomycin–Ceftazidime–Ciprofloxacin(*n* = 1PZ)
***Opric a[***22***]***	2004	Retrospective study (II)	328	304	304			P. acnes			Clindamycin Erythromycin Tetracycline Linezolyd Benzypenicillin Vancomycin
***Matse n[***23***]***	2015	Prospective study (I)	10	10	10	range 11–58	10/0	P. acnes			Ceftriaxone Vancomycin
***Parad a[***24***]***	2018	An on-line survey (V)	84	62	62			P. acnes			Cefazolin Vancomicin Clindamycin
***Al-Mayahi[***25***]***	2015	Case-control study (III)	2740	1167	2740	57		*S. aureus*			Quinolone Beta-Lactam Long-Acting Agent (Vancomycin, Teicoplanin, Ceftriaxone, Ertapenem)

The ineffectiveness of Cefazolin to eliminate the colonization of P. acnes was demonstrated in one study [20]. Parada et al. have made a cross-sectional study with surgeons specialized in shoulder arthroplasty, proposing an online survey to learn about the current protocols used for antibiotic prophylaxis [24]. The results of the study show that Cefazolin (90%), Vancomycin (50%) and Clindamycin (18%) are commonly used antibiotics [24]. Three studies define that preoperative antibiotics are not always able to eliminate *Propionibacterium* from the surgical field during shoulder arthroplasty [19, 22, 23]. Study outcomes are reported in Table 2.

**Table 2:** Study outcome

Author	Antibiotic dosage	Administration time	Mic μg/ml (mean)	Culture-negative	Culture-positive	Outcome
***Pérez-Priet o[***19***]***	Cefalosporin 2 g; Glycopeptide 1 g	30 and 60 min before surgery		8	20	Preoperative antibiotic prophylaxis does not eliminate suspected or confirmed cultures of PJI intraoperatively.
***Kong Ko h[***20***]***	Cefazolin 2 g; Gentamicin 3 mg/kg.	30 min before surgery	0.32	8	22	Cefazolin is not effective or sufficient to eliminate P. acnes colonies.
***Lut z[***21***]***	Clindamycin–Ofloxacin (*n* = 14 PZ) Rifampicin–Ofloxacin(*n* = 6PZ) Clindamycin–Rifampicin (*n* = 3PZ) Cloxacillin–Ofloxacin(*n* = 2) Rrifampicin–Pristinamycin (*n* = 2PZ) Ceftriaxone–pristinamycin(*n* = 1PZ) Cefepime–Ofloxacin (*n* = 1PZ) Reicoplanin–Rifampicin (*n* = 1PZ) Clindamycin–Fusidic Acid (*n* = 1PZ) Clindamycin monotherapy(*n* = 1PZ) Vancomycin–Ceftazidime–Ciprofloxacin(*n* = 1PZ)					Antibiotic prophylaxis is effective for preventing infection.
***Opric a[***22***]***	Clindamycin Erythromycin Tetracycline Linezolyd Benzypenicillin Vancomycin		< 0,064–64 > < 0,064- > 128 < 0,064–32 > < 0,25–2 > < 0,008-0,125 > < 0,25–2>			It was concluded that antimicrobial tested do not always eliminate p. acnes.
***Matse n[***23***]***	Ceftriaxone 2 g; Vancomycin 1 g	30 aminutes before surgery		7	3	In the shoulder arthroplasty, the preoperative prophylaxis is not always eradicated Propionibacterium from the operative field.
***Parad a[***24***]***	Cefazolin Vancomicin Clindamycin					In Propionibacterium infections during shoulder arthroplasty, there are no precise clinical guidelines.
***Al-Mayah i[***25***]***	Quinolone Beta-Lactam Long-Acting Agent (Vancomycin, Teicoplanin, Ceftriaxne Ertapenem)	1 h before surgery		26 (8%) 136 (43%) 33 (10%)	105 (4%) 570 (24%) 189 (8%)	Preoperative antibiotic prophylaxis, even in a single dose, is associated with negative results in culture.

### Study quality

EPHPP tool domain ratings indicate that seven studies did not report the reliability of the data collected and, therefore, were classified as poor quality (Table 3) [19–25].

**Table 3:** EPHPP quality assessment ratings

***Authors, year***	***Global rating***	***Selection Bias***	***Study Design***	***Confounders***	***Blinding***	***Data Collection Methods***	***Withdrawals and Drop-Outs***
***Pérez-Prieto, 2016[***19***]***	Weak	Weak	Strong	Moderate	Strong	Strong	Moderate
***Kong Koh, 2016[***20***]***	Weak	Weak	Strong	Moderate	Moderate	Strong	Moderate
***Lutz, 2005[***21***]***	Weak	Weak	Weak	Weak	Strong	Strong	Moderate
***Oprica, 2004[***22***]***	Weak	Strong	Weak	Weak	Strong	Strong	Moderate
***Matsen, 2015[***23***]***	Weak	Weak	Weak	Weak	Moderate	Strong	Moderate
***Al-Mayahi, 2015[***25***]***	Weak	Weak	Weak	Weak	Strong	Strong	Moderate
***Parada, 2018[***24***]***	Weak	Weak	Weak	Strong	Weak	Strong	Moderate

## Discussion

*P. Acnes* are microorganisms that populate the shoulder area where they found their natural site not only in superficial tissues but also in deep ones [15]. In the pre-operative period, 6 culture swabs were collected to be treated with calcium arginate. Some swabs were made on the surface at a maximum of 15 cm above the deltopectoral incision, others performed more deeply in the choracoid area. Three swabs were used to rule out pre-contamination and confirm the sterility of the operating room and analysis laboratories [20]. Moreover, a correlation between the male gender and the presence of *P. Acnes* has been demonstrated in 30 consecutive series of 30 patients undergoing primary shoulder arthroplasty. In fact, the hair of male gender in the shoulder area are known as important factors in colonization. The result expressed by the analyzed swabs showed that the male gender with the presence of hair is significantly correlated to the positive presence of P. acnes in the superficial area. Contrary in the female gender, there are no superficial or profound positives in P. acnes cultures [20].

The reduction of the surgical site infection rate is possible through the standardized administration of prophylactic antimicrobials, particularly in arthroplasty surgery. Due to its broad-spectrum, the use of cephalosporin is an unscientific approach to avoid shoulder PJI [24]. Furthermore, penicillin, known as very effective against P. Acnes, has shown good results as a preoperative antibiotic, but it is still not completely used [26]. Currently, the combination of an antimicrobial beta-lactam and cefazolin is considered the most appropriate preoperative prophylaxis due to their broad coverage spectrum, especially in shoulder arthroplasty. Cefazolin is active against a large number of infective organisms, skin flora and aerobic gram-positive [11]. Cefazolin is also active against bacteria as it is water-soluble and active bactericide capable of inhibiting cell wall biosynthesis bringing to bacterial lysis [11]. Furthermore, due to its good pharmacokinetics, it is able to quickly reach the site at the time of the incision, demonstrating effective bone, synovial and muscular penetration. Further benefits of Cefazolin are low cost, good safety profile and long half-life after intravenous administration [27]. Instead of Cefazolin, which is still the preferred antimicrobial drug, Clindamycin is recommended by the American Academy of Orthopaedic in patients with beta-lactam allergy and in those with methicillin-resistant *Staphylococcus aureus* [11]. Clindamycin has a higher minimum of bacterial concentration and a minimum concentration of biofilm eradication. However, it offers an important action against aerobic bacteria but not often against gram-negative bacteria. Clindamycin is a semi-synthetic antibiotic, better absorbed and with a greater antibacterial activity against some pathogens. It is active against numerous gram-positive aerobes and some protozoa and is not always active against aerobic tram-negatives [11, 28].

This systematic review showed that the optimal perioperative antimicrobial regimen is controversial. Although the use of antibiotic prophylaxis with a single drug is recommended by clinical guidelines, the increase in antibiotic-resistant infections may suggest that the choice to increase prophylactic antibiotic regimens may not be correct, improving the risk of expanding bacterial resistance.

The efficacy of the combination of Cefazolin and Clindamycin was studied. These drugs lead to potential damage such as acute postoperative renal damage and Clostridium infection and, therefore, the risk-benefit ratio must be carefully evaluated before performing a large-scale operation [7]. However, the use of combined prophylaxis between Cefazolin and Clindamycin in shoulder arthroplasty is increasing and, therefore, further research is needed to assess long-term effects [7].

The purpose of the preoperative administration of antimicrobials is to allow adequate tissue concentrations capable of eliminating the organisms that could occur before surgery. Therefore, the timing of administration and their dosage must be carefully studied to improve the effectiveness of prophylaxis. Single-shot antibiotic prophylaxis is normally sufficient to prevent the onset of bacteria but is not always capable of controlling P. Acnes [19]. No major reduction of P. Acnes was found on the surface and deep of the surgical wound layer after a careful prophylactic antiseptic protocol [20]. The effective treatment of the infection should include both medical and surgical therapy [21, 22]. Antibiotic treatment may not eliminate P. Acnes as it has proven insufficient in eradicating it [23]. The administration of antibiotic prophylaxis does not exclude contamination from P. Acnes, sometimes not administering antibiotics can guarantee a low rate of onset of P. Acnes [25]. As an increase in PJI has been reported when antibiotics are administered 60 min before the incision, current American Academy of Orthopedic Surgeons, Center for Disease Control and Surgical Care Improvement guidelines recommend the administration of antibiotic prophylaxis within 1 h of the surgical procedure; while the European guidelines report the administration of a single dose within 30 min of the incision [11]. In case of significant blood loss or prolonged surgery, additional doses of antibiotic will be required. In these cases, cefazolin should be repeated for 2–5 h during surgery, while clindamycin should be repeated every 3–6 h during surgery unless the patient shows altered renal parameters. Furthermore, the duration of administration of the antibiotic should not exceed 24 h after surgery since no benefit has been demonstrated in extending antimicrobial administration beyond 1 day after surgery [29].

For the prevention of PJI, the administration of preoperative antibiotic prophylaxis seems to be more effective than normal sterilization to minimize bacterial contamination, in particular from *Propionibacterium acnes*, before the incision [24]. Although guidelines are available for the administration of antibiotic prophylaxis to prevent PJI in shoulder replacement surgery, they are based on a limited number of randomized controlled trials that identify the efficacy of antimicrobial prophylaxis. The American Society of Health-System Pharmacists has demonstrated the effectiveness of cephalosporin in reducing PJI in surgical procedures [29]. The recommended dose of cefazolin in adults is 2 g, while for clindamycin it is 900 mg. The pharmacological characteristics of this antimicrobial class can be modified by the patient’s body weight, therefore its dosage must be specific for the patient [30].

This systematic review has a limitation represented by the small sample size of the included studies. Therefore, it is essential to define the concept of this contamination for the use of the necessary sterilization techniques to be used in the pre-operative phase [11]. Even though the differences in antibiotic prophylaxis between primary implantation and revision replacement may have considerable clinical relevance, it was not possible to perform an analysis since none of the seven studies included reported these data. Further researches are warranted to define the potential role of different antibiotic prophylaxis between primary and revision shoulder replacement.

## Conclusions

There is a lack of consensus in the administration of preoperative antibiotics to prevent infection in shoulder arthroplasty surgery. In this systematic review, we found that the optimal perioperative antimicrobial regimen is controversial. The clinical guidelines recommend the use of only one antibiotic as prophylaxis but considering the increase in the rates of antibiotic-resistant infections, the question arises whether antibiotic prophylaxis should be extended for adequate coverage. Moreover, our results suggest that the male population is more likely to have a higher concentration of *Propionibacterium acnes* in the axillary region and, therefore, its presence must be carefully controlled in the post-operative phase of shoulder arthroplasty.

Further research is needed to find more effective techniques for the prevention of PJIs, to define the preparation of the surgical site in order to minimize the possibility of contamination and identify adequate antibiotic prophylaxis taking into account the risks and benefits of individual drugs.

## Data Availability

All data generated or analysed during this study are included in this published article.

## Abbreviations

PJI: Periprosthetic joint infection
P. acnes: *Propionibacterium acnes*
EBM: Evidence Based Medicine
ICM: International Consensus Meeting
EPHPP: Effective Public Health Practice Project
